# Teacher agency in online teaching: contextual conditions and psychological adaptation experiences among international Chinese language teachers

**DOI:** 10.3389/fpsyg.2026.1953643

**Published:** 2026-10-19

**Authors:** Yi Wu

**Affiliations:** Faculty of International Education, Qingdao Hengxing University of Science and Technology, Qingdao, Shandong, China

**Keywords:** international Chinese language teachers, mixed methods, online teaching, psychological adaptation, teacher agency, teacher information literacy

## Abstract

**Introduction:**

Digital transformation has changed the conditions of teaching, requiring educators to adapt their instructional practices and respond to new professional demands. Teacher agency has been widely examined from ecological perspectives, whereas less attention has been paid to teachers' psychological adaptation experiences in digitally mediated teaching contexts. This study examines the associations between teacher agency and selected personal and contextual conditions among international Chinese language teachers engaged in online teaching and explores teachers' psychological adaptation experiences in relation to agency.

**Methods:**

The study used an explanatory sequential mixed-methods design. The quantitative phase involved a survey of 312 international Chinese language teachers with online or blended teaching experience, followed by a qualitative phase involving four purposively selected cases. Quantitative data were analyzed using descriptive statistics, confirmatory factor analysis, correlation analysis, and multiple regression analysis, while qualitative data were analyzed thematically.

**Results:**

The results showed that behavioral agency had the highest descriptive mean among the three dimensions of teacher agency. The regression analysis showed that teacher information literacy, school support, and student cooperation were independently associated with teacher agency, whereas colleague collaboration was not independently associated with agency when the four conditions were considered simultaneously. The full model accounted for 53.9% of the variance in teacher agency. The qualitative findings identified three recurring patterns in teachers' psychological adaptation experiences: emotional adaptation, professional reflection, and identity negotiation.

**Conclusion:**

This research builds on ecological perspectives of teacher agency by considering psychological adaptation as a qualitative explanatory lens for understanding teachers' experiences and responses in online teaching contexts. The findings highlight the relevance of social and institutional resources, as well as opportunities for reflection, identity development, and psychological support, to understanding teacher agency in online teaching.

## 1 Introduction

Digital transformation has reshaped educational practice beyond the simple adoption of technology, requiring teachers to reconsider their pedagogical practices, professional roles, and use of digital resources (Chen, 2022; Lee and Jeon, 2024). Teachers in digitally mediated environments must continually make pedagogical decisions and adjust their practices in response to changing instructional conditions (Ashton, 2022; Chen, 2022). These demands are particularly salient in language education, where teachers must manage technology use, interaction, learner engagement, and diverse instructional needs across digitally mediated contexts (Ashton, 2022; Chen, 2022). The idea of teacher agency is rooted in considering teachers' ability to make purposeful decisions that affect their professional practices in particular contexts. Teacher agency contrasts with views of teachers as passive recipients of educational reform and instead emphasizes their capacity for purposeful action within particular contexts (Biesta and Tedder, 2007; Emirbayer and Mische, 1998). Priestley et al. (2015) offer an ecological perspective in which teacher agency emerges through the interaction between teachers' personal capacities and the cultural, structural, and material conditions of their professional environments. This ecological perspective is particularly relevant to digitally mediated teaching, where teachers enact agency through ongoing interaction with technological affordances, professional experiences, and contextual conditions (Chen, 2022; Lee and Jeon, 2024). Although ecological research has increasingly examined the technological, institutional, and relational conditions of teacher agency, recent studies have also highlighted the relevance of teachers' emotions, identities, and professional interpretations in agency enactment (Lee and Jeon, 2024; Tao et al., 2024). Nevertheless, how teachers' subjective adaptation experiences complement evidence on personal and contextual conditions remains less clearly understood, particularly among international Chinese language teachers.

International Chinese language teachers work in multilingual, multicultural, and technology-mediated contexts that necessitate the constant modification of teaching techniques. However, the transition to online teaching has intensified these demands by introducing challenges related to technology use, reduced interaction, and changing instructional requirements (Ashton, 2022; Chen, 2022; Lee and Jeon, 2024). Although studies show that language teachers actively respond to these challenges through adaptation and reflection, less is known about how personal and contextual conditions are associated with teacher agency and how international Chinese language teachers experience psychological adaptation in online teaching contexts (Chen, 2022; Tao et al., 2024). Teachers facing similar professional challenges do not necessarily respond in the same way; they may interpret and adapt to these conditions through different emotional, reflective, and identity-related experiences. In this study, psychological adaptation refers to teachers' emotional, reflective, and identity-related responses to uncertainty and changing professional demands. Recent research has further shown that teachers' emotional experiences are closely intertwined with agency enactment in online teaching, highlighting the relevance of emotional responses to how teachers experience and respond to changing instructional conditions (Tao et al., 2024). Besides, Schön's (1983) concept of reflective practice states that teachers gain knowledge through constant reflection. Considering psychological adaptation alongside ecological perspectives on teacher agency may therefore provide a complementary lens for understanding how teachers interpret and respond to their professional conditions (Priestley et al., 2015; Tao et al., 2024).

The overall purpose of this study is to develop a more comprehensive understanding of teacher agency among international Chinese language teachers engaged in online teaching by examining its associations with selected personal and contextual conditions and exploring teachers' psychological adaptation experiences in relation to agency. To achieve this purpose, an explanatory sequential mixed-methods design was employed. The quantitative phase examined the level and pattern of teacher agency and its associations with selected personal and contextual conditions, while the subsequent qualitative phase explored teachers' psychological adaptation experiences to provide contextualized explanations of the quantitative patterns. The quantitative findings informed the focus of the qualitative inquiry by identifying patterns and associated conditions that required further contextual explanation. The two strands were subsequently integrated at the interpretation stage, where qualitative accounts were used to elaborate, contextualize, and, where relevant, contrast with the quantitative findings. Psychological adaptation was therefore used as a qualitative explanatory lens rather than as a quantitatively tested mediator or causal mechanism. Accordingly, RQ1 and RQ2 were addressed in the quantitative phase, whereas RQ3 was addressed in the subsequent qualitative phase. RQ3 built on the quantitative findings by exploring how teachers described their psychological adaptation experiences in relation to the agency patterns and associated personal and contextual conditions identified in the quantitative phase. The findings across the three research questions were subsequently integrated at the interpretation stage to provide a contextualized mixed-methods understanding of teacher agency in online teaching. Based on the identified research gaps and the proposed conceptual framework (Figure 1), this study addresses the following research questions:

**Figure 1:**
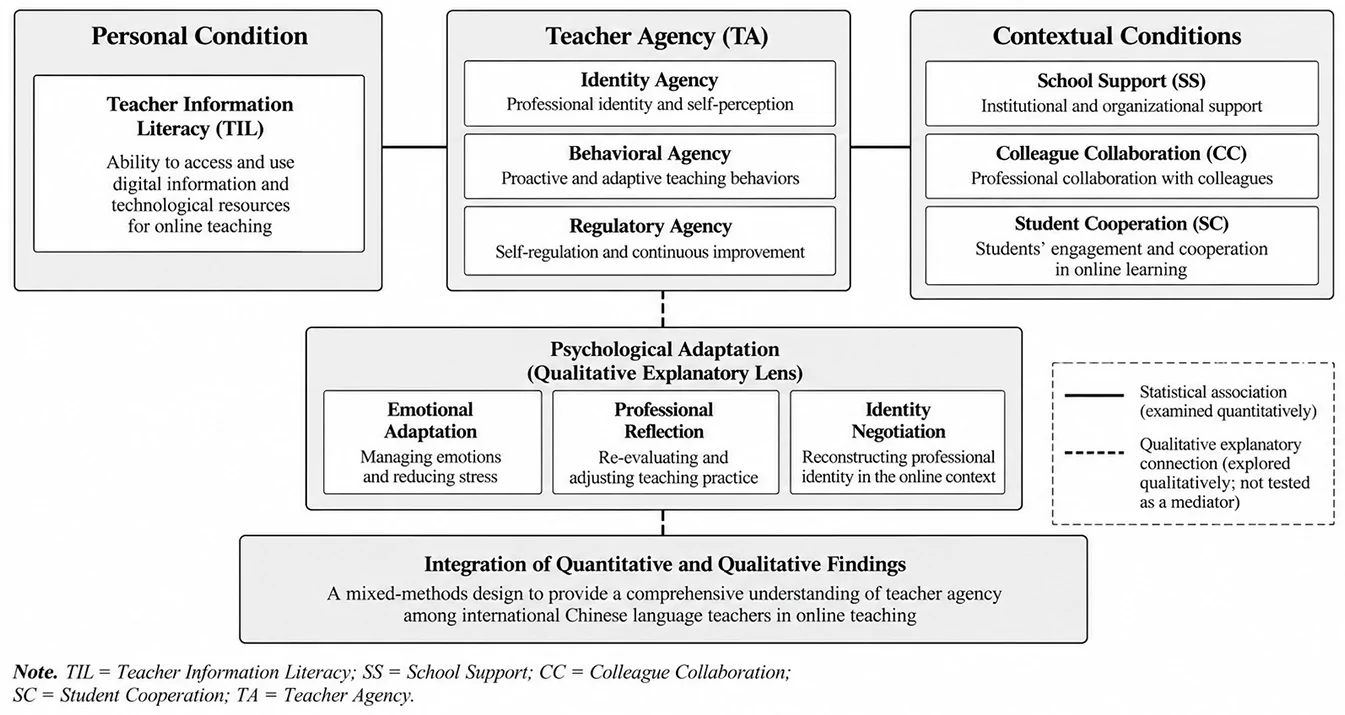
Conceptual framework of the mixed-methods study.

RQ1. What is the current level and pattern of teacher agency among international Chinese language teachers engaged in online teaching?

RQ2. What personal and contextual conditions are associated with teacher agency in online teaching?

RQ3. How do international Chinese language teachers describe their psychological adaptation experiences in relation to teacher agency in online teaching?

## 2 Literature review

### 2.1 Ecological perspectives of teacher agency

Teacher agency is a well-known concept for understanding how teachers actively engage with professional change. Teacher agency can be broadly understood as individuals' capacity to act purposefully within social contexts. Emirbayer and Mische (1998) conceptualized agency as temporally embedded, with action shaped by orientations toward the past, present, and future. Current educational research has increasingly shifted from individual-focused conceptions toward an ecological view of teacher agency. According to Biesta and Tedder (2007), agency should be regarded as something gained in the relations of people with their environment rather than something individual. From this perspective, teachers realize their agency by interpreting situational requirements with the help of available resources, gained information, and their ability to make professional decisions in certain situations. Expanding on this ecological point of view, Priestley et al. (2015) suggest that teacher agency emerges out of the interplay of personal capacities, professional contexts, and cultural resources. Teacher agency is thus not a static personal feature but rather an evolving process created through constant negotiation between teachers and their settings. Online education has led teachers to make constant decisions about their teaching methods, technology use, work with learners as well as professional development (Chen, 2022; Lee and Jeon, 2024). Thus, teacher agency provides a useful perspective for understanding how teachers respond to challenges, adjust their professional practices, and develop responses to educational change. Research in language education has further shown that teacher agency is manifested in how teachers respond to professional challenges, develop their practices, and negotiate changing educational demands (Tao and Gao, 2021; Ashton, 2022; Lee and Jeon, 2024). However, although ecological perspectives explain how agency emerges through the interplay between teachers and their environments, further attention is needed to teachers' subjective experiences of interpreting and responding to changing professional conditions, particularly in digitally mediated teaching contexts (Tao et al., 2024).

### 2.2 Teacher agency in digital teaching contexts

Digital transformation generates new demands for teachers due to the fact that teachers need to reshape their teaching methods and develop new forms of interaction with the learners. The online teaching has brought uncertainty due to the use of technology, less interaction between people and new expectations concerning the learning process. Understanding teacher agency is therefore important for examining how teachers respond to changes in digital education. According to various studies, successful online teaching requires teachers to rethink their pedagogical methods, redesign their activities, and find ways of engaging students. Hodges et al. (2020) pointed out that online teaching calls for planning and learning design instead of simply providing teaching that is effective for face-to-face learning. Likewise, Rapanta et al. (2020) stressed the importance of having teacher presence, instructional design, and active facilitation in online learning. Research on language teachers further indicates that digitally mediated teaching requires continuous pedagogical adjustment and agentic decision-making (Chen, 2022; Lee and Jeon, 2024). MacIntyre et al. (2020) reported that language teachers encountered emotional and professional challenges during their transition to online teaching and that their coping strategies were associated with their responses to these instructional demands. According to the results of Cutri et al. (2020), online teaching also changed the professional roles of teachers, some of whom had to modify their emotional experience and understanding of the instructional process. From a teacher agency viewpoint, online classrooms offer both limitations and possibilities. Some elements of the technology come with limitations, including extra work and limited communication. On the other hand, digital contexts provide the possibility of trying new methods for teaching and reconstructing professional practice. The current research states that the agency of language teachers involves constant making of decisions, adaptation, and reconstructing of professional practice during the process of digital transformation (Hu and Shen, 2024). The current set of studies has focused mainly on adaptation by teachers in online teaching. However, less is known about how evidence on personal and contextual conditions can be considered alongside teachers' psychological adaptation experiences in understanding teacher agency, particularly among international Chinese language teachers. Further research is therefore needed to examine teacher agency in relation to these conditions while also exploring how teachers describe their psychological adaptation experiences in digitally mediated teaching.

### 2.3 Personal and contextual conditions and psychological adaptation in teacher agency

Ecological perspectives emphasize that teacher agency does not occur in isolation but is situated within the interplay between personal capacities and contextual conditions (Biesta and Tedder, 2007; Priestley et al., 2015). Consequently, the development of agency is not regarded as an innate personal ability but as a process manifested in the actions of teachers who make use of various professional resources, social relations, and institutional structures. Among these personal capacities, teacher information literacy (TIL) represents a relevant personal condition in digitally mediated educational environments. Information literacy is not limited to the ability of an individual to use the digital tools but also means that the individual uses information for professional purposes (Falloon, 2020; Redecker, 2017). It has been noted that teachers' digital competence plays a key role in creating technology-enhanced learning experiences (Lucas et al., 2021). Another contextual condition associated with teacher agency is institutional support. Supportive educational environments provide teachers with the necessary resources, opportunities for their professional development, and institutional acknowledgment. All these things can promote teachers' adaptation and innovative behavior (Opfer and Pedder, 2011). Teacher agency is also closely related to interpersonal relationships. In online teaching, student participation and feedback can provide teachers with information for evaluating and adjusting their teaching practices. Collaboration with colleagues may also provide opportunities for knowledge exchange, professional support, and collaborative problem solving (Vangrieken et al., 2015). While the context plays an important role in the teaching profession, it by no means explains the differences in the responses of various teachers to the same educational challenges. Teachers may interpret and respond to similar professional conditions in different ways based on their experiences. Psychological adaptation may therefore provide a complementary lens for understanding how teachers experience, interpret, and respond to personal and contextual conditions in relation to their agency.

According to Lazarus and Folkman (1984), psychological adaptation involves the means people use in regulating their emotions, reconstructing their understandings, and changing their behaviors in response to situations. In educational contexts, such adaptation may involve responding to uncertainty, reflecting on professional practice, and negotiating professional identity. In the present study, these experiences are explored qualitatively and are not modeled as a mediating variable between personal or contextual conditions and teacher agency.

In online teaching contexts, psychological adaptation may be reflected in teachers' emotional responses, professional reflection, and identity negotiation. Recent research has shown that teachers' emotional experiences are closely intertwined with agency enactment in online teaching, as teachers interpret and respond to changing professional demands (Tao et al., 2024). Professional reflection provides a chance for teachers to analyze their experiences, modify their teaching approaches, and acquire new knowledge (Schön, 1983). Identity negotiation is a process during which teachers redefine their professional meanings based on the changes in the education environment (Beijaard et al., 2004).

Considering psychological adaptation alongside ecological perspectives may provide a complementary understanding of how teachers experience agency in changing professional contexts. From this perspective, personal and contextual conditions can be examined in relation to teacher agency, while psychological adaptation provides a qualitative lens for exploring how teachers experience, interpret, and respond to these conditions.

## 3 Methodology

### 3.1 Research design

The present study applied an explanatory sequential mixed-methods approach to examine teacher agency among international Chinese language teachers in online teaching contexts. This design was selected because the study aimed not only to examine the level of teacher agency and its associations with selected personal and contextual conditions but also to explore teachers' psychological adaptation experiences in relation to teacher agency.

The study followed the framework outlined by Creswell and Plano Clark (2018), completing two stages in succession. In the quantitative phase, questionnaire data were collected from 312 international Chinese language teachers to examine the overall level and dimensions of teacher agency and its associations with selected personal and contextual conditions. Descriptive statistics, confirmatory factor analysis (CFA), correlation analysis, and multiple regression analysis were performed.

The quantitative findings identified associations between teacher agency and selected personal and contextual conditions but could not explain how teachers experienced and interpreted these conditions in their professional practice. Therefore, the qualitative phase was used to explore teachers' psychological adaptation experiences in relation to their experiences of teacher agency in online teaching. The qualitative findings provided insight into how teachers described emotional adaptation, professional reflection, and identity negotiation in response to the challenges of online teaching. The two strands were integrated at the interpretation stage to examine how the qualitative findings complemented, contextualized, or diverged from the quantitative patterns. Psychological adaptation was treated as a qualitative explanatory lens rather than as a quantitatively tested mediator between personal or contextual conditions and teacher agency.

The overall sequence and integration of the explanatory sequential mixed-methods design are illustrated in Figure 2.

**Figure 2:**
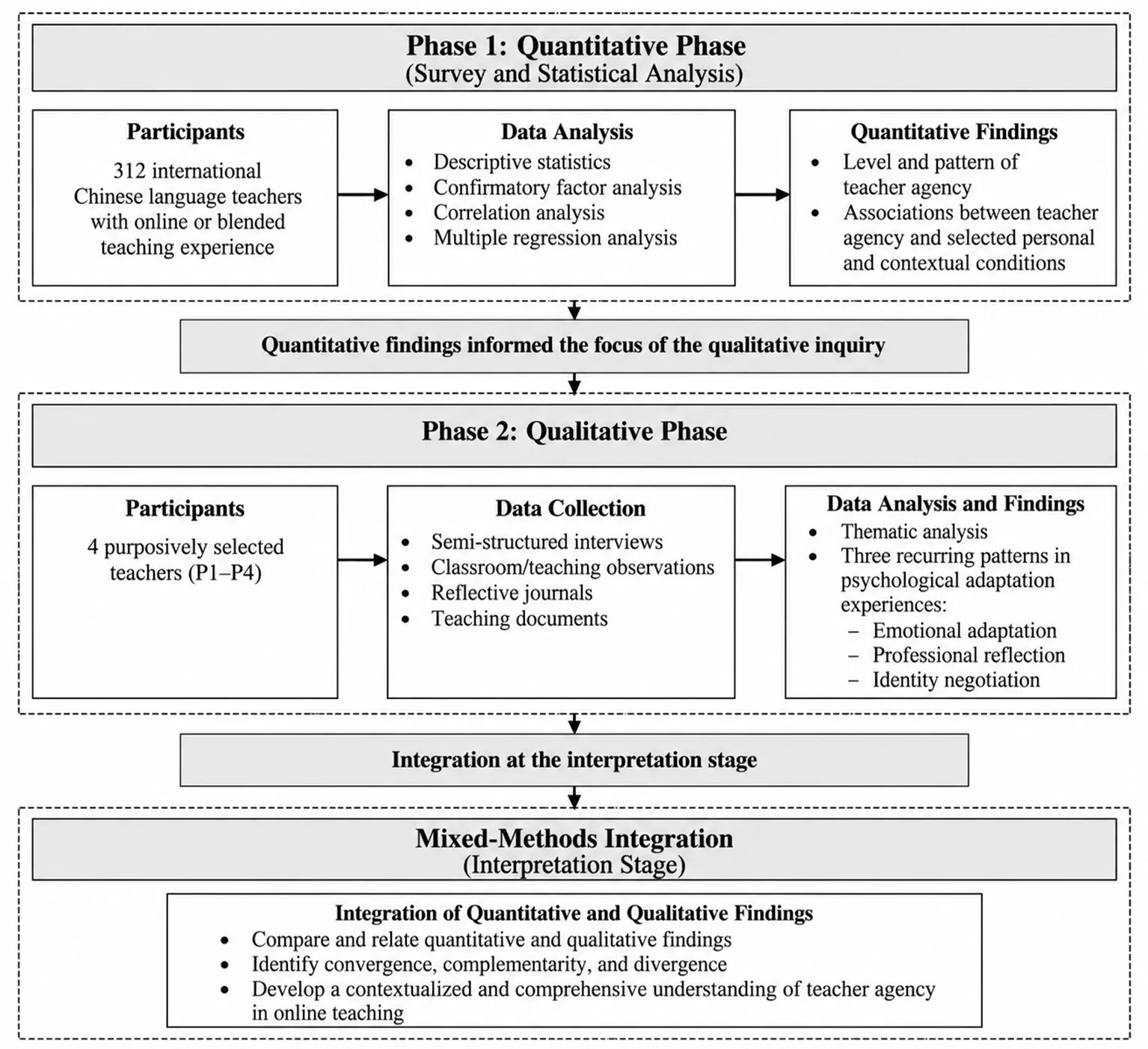
Explanatory sequential mixed-methods design and integration process.

### 3.2 Research context

The study was conducted in the context of online and blended international Chinese language teaching. Participants were teachers engaged in international Chinese language education who had at least one semester of online or blended teaching experience. Their teaching contexts varied in terms of institutional settings, technological conditions, and access to professional support. Most participants reported conducting online teaching in China (*n* = 295, 94.55%), while a smaller proportion reported teaching from overseas locations (*n* = 17, 5.45%). Accordingly, the term “international Chinese language teachers” in this study refers to teachers working in the professional field of international Chinese language education rather than to a geographically representative international sample.

### 3.3 Participants and data collection

In the quantitative phase, 312 international Chinese language teachers participated in the study. Eligible participants had at least one semester of online or blended teaching experience. The sample included 148 male (47.44%) and 164 female (52.56%) teachers. In terms of age, 71 participants (22.76%) were under 30 years old, 131 (41.99%) were aged 31–45, and 110 (35.26%) were aged 46 or above. Regarding teaching experience, 123 participants (39.42%) had less than 5 years of experience, 159 (50.96%) had 5–10 years, and 30 (9.62%) had more than 10 years of teaching experience. Further demographic characteristics are presented in Table 1.

**Table 1:** Demographic characteristics of the quantitative participants.

Characteristic	Category	*N*	%
Gender	Male	148	47.44
	Female	164	52.56
Age	< 30 years	71	22.76
	31–45 years	131	41.99
	≥46 years	110	35.26
Teaching experience	< 5 years	123	39.42
	5–10 years	159	50.96
	>10 years	30	9.62
Education	College	26	8.33
	Bachelor's degree	169	54.17
	Master's degree	90	28.85
	Doctoral degree	27	8.65
Professional title	Professor	23	7.37
	Associate Professor	55	17.63
	Lecturer	139	44.55
	Teaching Assistant	95	30.45
Online teaching location	China	295	94.55
	Overseas	17	5.45

For the qualitative phase, four teachers were purposively selected from survey participants who had agreed to further participation. Participant selection sought variation in age, gender, educational background, teaching experience, and teaching context so that different experiences of online teaching could be examined. The four participants included two women and two men: one was born in the 1970s, two in the 1980s, and one in the 1990s. Two held doctoral degrees and two held master's degrees. Three were teaching in China, while one was teaching in Malaysia. Pseudonyms P1–P4 were used throughout the analysis to protect participant confidentiality. The demographic and professional characteristics of the four qualitative participants are summarized in Table 2.

**Table 2:** Characteristics of the qualitative participants.

Participant	Career stage/Teaching experience	Teaching context	Relevant professional background
P1	Early-career teacher; approximately 4 years	Online Chinese language teaching	International Chinese language teacher with online teaching experience
P2	Experienced teacher	China-based international Chinese language teaching	Previous overseas Chinese language teaching experience
P3	Experienced teacher; approximately 16 years	University-based international Chinese language teaching in China	Associate professor with extensive teaching and research experience
P4	Experienced teacher; approximately 10 years	International Chinese language teaching in Malaysia	Extensive overseas and online teaching experience

Six teachers were initially approached for the qualitative phase, and four were retained for in-depth case analysis based on their continued participation, availability of sufficiently rich case materials, and variation across participant characteristics and teaching contexts.

The qualitative cases were intended to provide information-rich accounts for the explanatory phase of the mixed-methods design rather than to achieve statistical representativeness.

### 3.4 Data collection

In the quantitative phase, questionnaire data were collected from international Chinese language teachers with at least one semester of online or blended teaching experience. Before the main data collection, the questionnaire was piloted with 35 teachers to assess its clarity and suitability for the target population. The final quantitative sample consisted of 312 valid responses. Participation was voluntary, and participants were informed of the purpose of the study before completing the questionnaire.

The qualitative phase was conducted from May to November 2024. Data were collected through semi-structured interviews, classroom observations, teaching journals and reflective materials, and supplementary teaching documents. Each of the four participants took part in three successive semi-structured interviews. The first interview focused on participants' online teaching experiences, professional changes, and teaching beliefs; the second explored personal and contextual conditions, professional choices, and perceived constraints; and the third followed up on issues emerging from the earlier interviews and explored participants' interpretations of their experiences and professional goals.

Interviews were conducted at locations selected by the participants and were audio-recorded with their consent. The recordings were transcribed for analysis, with relevant contextual information, such as notable emotional expressions or non-verbal responses, recorded where appropriate. Classroom observations were also conducted to provide contextual information about participants' online teaching practices and interactions. Teaching journals and other reflective materials were used to complement the interview and observation data.

The use of multiple qualitative data sources enabled comparison across interviews, observations, and reflective materials, thereby providing a richer contextual basis for interpreting the four cases.

### 3.5 Measure

The questionnaire measures were adopted from the instrument developed by Wang and Xing (2022) for international Chinese language teachers and were used with the authors' permission. The original items were retained without modification. The questionnaire included teacher agency and four personal or contextual conditions: school support (SS), colleague collaboration (CC), student cooperation (SC), and teacher information literacy (TIL). Teacher information literacy was treated as a personal condition, whereas school support, colleague collaboration, and student cooperation were treated as contextual conditions. All items were rated using five-point response scales.

The teacher agency scale contained 29 items across three dimensions: identity agency (11 items), behavioral agency (11 items), and regulatory agency (7 items). Higher scores indicated higher levels of perceived teacher agency. In the present sample, Cronbach's α was 0.928 for identity agency, 0.917 for behavioral agency, 0.877 for regulatory agency, and 0.945 for the overall teacher agency scale.

School support (SS), colleague collaboration (CC), and student cooperation (SC) were assessed using three, two, and three items, respectively. These measures captured teachers' perceptions of institutional support, professional collaboration with colleagues, and students' cooperation in online teaching. Cronbach's α was 0.830 for SS, 0.786 for CC, and 0.805 for SC.

Teacher information literacy (TIL) was assessed using the two original items from Wang and Xing (2022), without modification. The items focused on teachers' perceived ability to access and use digital information and technological resources for online teaching. TIL was treated as a focused personal capacity related to the use of information and technological resources in teaching rather than as a comprehensive measure of digital competence. Cronbach's α for the two-item measure was 0.766.

The measurement structure was evaluated using confirmatory factor analysis (CFA). Further evidence concerning construct reliability and validity is reported with the measurement-model results. The construct reliability and convergent validity results are presented in Table 3.

**Table 3:** Construct reliability and convergent validity.

Construct	Items	Cronbach's α	Factor loading range	CR	AVE
Identity agency (IA)	11	0.928	0.813–0.820	—	—
Behavioral Agency (BA)	11	0.917	0.794–0.850	—	—
Regulatory Agency (RA)	7	0.877	0.738–0.820	—	—
Teacher Agency (TA)	29	0.945	—	0.844	0.643
School Support (SS)	3	0.830	0.768–0.813	0.830	0.619
Colleague Collaboration (CC)	2	0.786	0.775–0.837	0.788	0.650
Student Cooperation (SC)	3	0.805	0.713–0.815	0.806	0.581
Teacher Information Literacy (TIL)	2	0.766	0.774–0.807	0.769	0.625

IA, Identity agency; BA, Behavioral Agency; RA, Regulatory Agency; TA, Teacher Agency; SS, School Support; CC, Colleague Collaboration; SC, Student Cooperation; TIL, Teacher Information Literacy; CR, Composite Reliability; AVE, Average Variance Extracted.

### 3.6 Data analysis

Quantitative data were analyzed using SPSS 26.0 and AMOS 24.0. Descriptive statistics were first calculated to summarize the levels and patterns of teacher agency and the personal and contextual conditions examined in the study. The measurement structure was subsequently evaluated using confirmatory factor analysis (CFA). Internal consistency was assessed using Cronbach's alpha, while composite reliability (CR) and average variance extracted (AVE) were examined as additional indicators of construct reliability and convergent validity. Discriminant validity was evaluated by comparing the square root of the AVE for each construct with the correlations between constructs. Pearson correlation analysis was then conducted to examine bivariate associations between teacher agency and the four personal and contextual conditions.

A simultaneous multiple linear regression analysis was conducted with overall teacher agency as the dependent variable. Teacher information literacy, school support, colleague collaboration, and student cooperation were entered simultaneously as predictors. This approach was used to estimate the unique association of each condition with teacher agency while accounting for the other conditions in the model. Multicollinearity was assessed using variance inflation factors (VIFs), and the Durbin–Watson statistic was examined as an indicator of independence of residuals. Regression coefficients, standard errors, standardized coefficients, 95% confidence intervals, and significance levels were reported for all four predictors, including non-significant predictors.

At the mixed-methods level, the study combined deductive and inductive reasoning. The quantitative phase was primarily deductive, examining predefined constructs and associations derived from the conceptual framework. The qualitative phase incorporated an inductive orientation, in which codes, categories, and themes were developed through repeated engagement with the qualitative materials, while remaining guided by the broader research questions and sensitizing concepts of the study. Qualitative data were analyzed using thematic analysis following the general procedures described by Braun and Clarke (2006). Interview transcripts, reflective materials, observation records, and other relevant case materials were repeatedly read to achieve familiarity with each participant's experiences. Initial codes were generated from segments relevant to teachers' online teaching experiences, agency, contextual conditions, and psychological adaptation. Related codes were subsequently grouped into broader categories and candidate themes. Themes were reviewed within and across the four cases and refined through comparison with the original qualitative materials. The analysis ultimately focused on recurring patterns concerning emotional adaptation, professional reflection, and identity negotiation.

To strengthen the credibility and trustworthiness of the qualitative interpretation, data-source triangulation was employed by comparing evidence across interviews, reflective materials, observations, and supplementary case documents. This triangulation allowed interpretations to be checked across multiple forms of qualitative evidence rather than relying on a single data source. Within-case analysis was followed by cross-case comparison to identify both recurring patterns and differences among participants. Particular attention was paid to instances that did not fully conform to the dominant patterns so that variation across the four cases was retained in the interpretation. Because qualitative coding necessarily involved researcher interpretation, themes were repeatedly checked against the original case materials to reduce the risk of interpretations becoming detached from participants' accounts.

Integration occurred during the interpretation stage. Quantitative findings concerning the associations between teacher agency and the four personal and contextual conditions were compared with qualitative evidence concerning how teachers experienced and responded to these conditions in their professional practice. The qualitative findings were used to contextualize and elaborate on the quantitative patterns rather than to test a mediating mechanism. Particular attention was given to areas of convergence, complementarity, and divergence between the two strands.

### 3.7 Ethical considerations

The study was conducted in accordance with established ethical principles for research involving human participants. Formal ethical approval was waived by the Ethics Committee of Qingdao Hengxing University of Science and Technology because the study involved adult teachers, voluntary participation, and low-risk questionnaire and interview procedures. Participants were informed of the purpose of the study, the voluntary nature of participation, and their right to withdraw before taking part in the study. Consent was obtained prior to participation. Identifying information was removed or anonymized during data processing and reporting, and pseudonyms (P1–P4) were used for the qualitative participants to protect confidentiality.

## 4 Results

### 4.1 Measurement model and construct reliability and validity

The measurement model was evaluated using confirmatory factor analysis (CFA). The model demonstrated an acceptable overall fit to the data: χ^2^/df = 1.239, RMR = 0.031, GFI = 0.945, AGFI = 0.925, NFI = 0.945, IFI = 0.989, TLI = 0.986, CFI = 0.989, and RMSEA = 0.028.

The standardized factor loadings were generally satisfactory. For the higher-order teacher agency construct, the standardized loadings were 0.827 for identity agency, 0.776 for behavioral agency, and 0.800 for regulatory agency. For the four personal and contextual condition measures, standardized item loadings ranged from 0.768 to 0.813 for school support, from 0.775 to 0.837 for colleague collaboration, from 0.713 to 0.815 for student cooperation, and from 0.774 to 0.807 for teacher information literacy.

Composite reliability values were 0.844 for teacher agency, 0.830 for school support, 0.788 for colleague collaboration, 0.806 for student cooperation, and 0.769 for teacher information literacy. The corresponding AVE values were 0.643, 0.619, 0.650, 0.581, and 0.625, respectively. These results provided additional evidence of construct reliability and convergent validity.

Discriminant validity was also examined by comparing the square root of the AVE for each construct with the inter-construct correlations. The square-root AVE values were 0.802 for teacher agency, 0.787 for school support, 0.806 for colleague collaboration, 0.762 for student cooperation, and 0.791 for teacher information literacy. These values exceeded the corresponding inter-construct correlations, supporting the discriminant validity of the constructs. Internal consistency was satisfactory, with Cronbach's alpha coefficients ranging from 0.766 to 0.945 across the study measures.

As shown in Table 4, the square-root AVE values exceeded the corresponding inter-construct correlations, supporting the discriminant validity of the constructs.

**Table 4:** Discriminant validity of the study constructs.

Construct	SS	CC	SC	TIL	TA
SS	0.787				
CC	0.512	0.806			
SC	0.664	0.621	0.762		
TIL	0.541	0.436	0.574	0.791	
TA	0.742	0.531	0.746	0.742	0.802

Diagonal values are the square roots of AVE; off-diagonal values are inter-construct correlations. SS, School Support; CC, Colleague Collaboration; SC, Student Cooperation; TIL, Teacher Information Literacy; TA, Teacher Agency.

The CFA and subsequent substantive analyses were conducted using the same sample of 312 participants; therefore, the measurement-model results should be interpreted as evidence of model fit within the present sample rather than as an independent cross-sample validation.

### 4.2 Descriptive results for teacher agency

The overall mean score for teacher agency was 3.45 (*SD* = 0.685). Among the three dimensions, behavioral agency showed the highest mean score (*M* = 3.56, *SD* = 0.79), followed by regulatory agency (*M* = 3.48, *SD* = 0.82) and identity agency (*M* = 3.33, *SD* = 0.86).

Paired-sample comparisons indicated that identity agency was significantly lower than behavioral agency, *t* = −5.137, *p* < 0.001, and regulatory agency, *t* = −3.323, *p* = 0.001. The difference between behavioral agency and regulatory agency was not statistically significant, *t* = 1.927, *p* = 0.055. These findings describe variation across the three dimensions of teacher agency within the present sample. Descriptive statistics for teacher agency and the personal and contextual conditions are presented in Table 5.

**Table 5:** Descriptive statistics of teacher agency and personal and contextual conditions.

Variable	*N*	Minimum	Maximum	*M*	*SD*
Teacher agency	312	1.38	4.62	3.45	0.685
Identity agency	312	1.00	5.00	3.33	0.86
Behavioral agency	312	1.36	5.00	3.56	0.79
Regulatory agency	312	1.14	5.00	3.48	0.82
School support	312	1.00	5.00	3.31	0.931
Colleague collaboration	312	1.00	5.00	3.28	0.999
Student cooperation	312	1.33	5.00	3.55	0.903
Teacher information literacy	312	1.00	5.00	3.65	0.982

### 4.3 Associations between teacher agency and personal and contextual conditions

Pearson correlation analyses showed that overall teacher agency was positively associated with all four conditions examined in the study. Teacher agency was positively correlated with school support (*r* = 0.595, *p* < 0.001), colleague collaboration (*r* = 0.417, *p* < 0.001), student cooperation (*r* = 0.595, *p* < 0.001), and teacher information literacy (*r* = 0.579, *p* < 0.001). Among these bivariate associations, school support and student cooperation showed the largest correlations with teacher agency, followed closely by teacher information literacy, whereas colleague collaboration showed a comparatively smaller positive correlation. The complete correlation matrix is presented in Table 6.

**Table 6:** Pearson correlations among teacher agency and personal and contextual conditions.

Variable	M	SD	1	2	3	4	5
1. Teacher agency	3.45	0.685	—				
2. School support	3.31	0.931	0.595^**^	—			
3. Colleague collaboration	3.28	0.999	0.417^**^	0.415^**^	—		
4. Student cooperation	3.55	0.903	0.595^**^	0.541^**^	0.495^**^	—	
5. Teacher information literacy	3.65	0.982	0.579^**^	0.432^**^	0.338^**^	0.446^**^	—

^**^p < 0.01.

### 4.4 Multiple regression analysis

A simultaneous multiple regression analysis was conducted to examine the unique associations of teacher information literacy, school support, colleague collaboration, and student cooperation with overall teacher agency. The overall regression model was statistically significant, F(4, 307) = 89.59, *p* < 0.001, explaining 53.9% of the variance in teacher agency (*R*^2^ = 0.539, adjusted *R*^2^ = 0.533).

Teacher information literacy showed the largest standardized coefficient (β = 0.314, *p* < 0.001), followed by school support (β = 0.290, p < 0.001) and student cooperation (β = 0.269, *p* < 0.001). Colleague collaboration did not show a statistically significant unique association with teacher agency when the other three conditions were included in the model (β = 0.057, *p* = 0.213).

The VIF values ranged from 1.349 to 1.708, indicating no substantial multicollinearity among the predictors. The Durbin–Watson statistic was 1.989. The complete regression model, including unstandardized coefficients, standard errors, standardized coefficients, 95% confidence intervals, and the non-significant coefficient for colleague collaboration, is reported in Table 7.

**Table 7:** Simultaneous multiple regression predicting teacher agency.

Predictor	B	SE	β	t	*P*	95% CI for B	VIF
School support	0.214	0.036	0.290	5.999	< 0.001	[.144,0.284]	1.556
Colleague collaboration	0.039	0.031	0.057	1.249	0.213	[-0.023,0.101]	1.396
Student cooperation	0.204	0.038	0.269	5.313	< 0.001	[.129,0.280]	1.708
Teacher information literacy	0.219	0.031	0.314	6.980	< 0.001	[.158,0.281]	1.349

*N* = 312. Dependent variable = overall teacher agency. *R*^2^ = 0.539, adjusted *R*^2^ = 0.533, F(4, 307) = 89.59, *p* < 0.001; Durbin–Watson = 1.989.

### 4.5 Qualitative findings on psychological adaptation experiences

The qualitative analysis identified three recurring patterns in teachers' psychological adaptation experiences in relation to their agency in online teaching: emotional adaptation, professional reflection, and identity negotiation. These themes did not represent a tested psychological mechanism; rather, they reflected how participants described interpreting and responding to changing demands in their online teaching practices. A summary of the three themes, illustrative codes, and representative participant quotations is presented in Table 8.

**Table 8:** Summary of qualitative findings.

Theme	Illustrative codes	Representative participant quotation
Emotional adaptation	Anxiety and uncertainty; preparation and emotional adjustment; pressure and motivation	P1: “*Before each lesson, I felt really nervous and scared. Even though I knew a lot about the topics, I wasn't sure I could explain them well to my students*.”
Professional reflection	Reviewing teaching experiences; reconsidering instructional choices; adjusting teaching practice	P3: “*After each lesson, I would think about which place could be perfected..*.”
Identity negotiation	Professional uncertainty; questioning career suitability; professional competence and recognition	P2: “*I could not see the results even after I worked hard, and I could not be fully devoted to teaching, so I once suspected that I was not suitable for this career*.”

#### 4.5.1 Emotional adaptation

Emotional adaptation was evident in participants' accounts of uncertainty, anxiety, pressure, and their efforts to manage these experiences during online teaching. For example, P1 described considerable nervousness before teaching online: “*Before each lesson, I felt really nervous and scared. Even though I knew a lot about the topics, I wasn't sure I could explain them well to my students.”* P1 responded by rehearsing lessons in advance and reported that greater preparation gradually reduced this uncertainty: “*Once you have a good idea, the nervousness disappears naturally.”*

However, participants did not describe pressure in uniformly negative terms. P4, for example, viewed a certain degree of pressure as potentially motivating: “*When there is pressure, one feels a bit anxious, but if there is no pressure, one feels very unmotivated and self-blame arises again.”* These contrasting accounts suggest that emotional adaptation involved not simply reducing negative emotions but negotiating different responses to professional demands.

#### 4.5.2 Professional reflection

Professional reflection emerged as another recurring pattern in participants' accounts. Participants described reviewing their teaching experiences, reconsidering instructional choices, and adjusting teaching materials and activities in response to online teaching demands. P3, for example, explained that reflection became meaningful when it was grounded in concrete teaching experiences: “*I heard some teachers say that you should have some reflections after each lesson... But if the lesson is designed well and you have strong feelings, you will want to write about them*.” P3 also described routinely reviewing lessons: “*After each lesson, I would think about which place could be perfected...”*

Reflection was also connected with participants' attempts to improve practice through professional inquiry. P4 described the relationship between research and teaching reflection as follows: “*Research helps me to record my reflection on teaching and is also a spur to keep exploring in practice.”* Across these accounts, reflection was described as part of an ongoing process of reconsidering and adjusting professional practice rather than as a single response to technological change.

#### 4.5.3 Identity negotiation

Identity negotiation was reflected in how participants reconsidered their professional roles, expectations, and sense of competence in response to changing teaching experiences. P2 described a period of professional uncertainty when sustained effort did not appear to produce the expected outcomes: “*I could not see the results even after I worked hard, and I could not be fully devoted to teaching, so I once suspected that I was not suitable for this career.”* P2 subsequently sought advice and reconsidered professional priorities, illustrating how professional identity could be questioned and renegotiated during periods of uncertainty.

Other participants described identity in relation to professional responsibility and recognition. P4 emphasized the importance of demonstrating teaching competence in establishing trust with students, noting that “*to gain students' trust, the first thing is still for them to affirm your teaching ability.”* These accounts illustrate variation in identity-related experiences, ranging from questioning one's suitability for teaching to reinforcing a sense of professional competence and responsibility.

Across the four cases, psychological adaptation was therefore characterized by overlapping emotional, reflective, and identity-related experiences. The prominence and meaning of these experiences varied across participants. Some accounts emphasized anxiety and uncertainty, whereas others highlighted motivation, professional reflection, or a strengthened sense of professional responsibility. The qualitative findings thus provide contextualized accounts of how teachers experienced and responded to changing professional conditions rather than evidence of a uniform psychological pathway.

### 4.6 Integration of quantitative and qualitative findings

Integration of the quantitative and qualitative findings revealed both convergence and complementarity across the two strands. The quantitative phase followed a primarily deductive logic by examining predefined associations between teacher agency and the selected personal and contextual conditions. The qualitative phase incorporated an inductive orientation by developing themes from participants' accounts and related case materials, while remaining guided by the broader research questions and sensitizing concepts of the study. At the integration stage, the quantitatively identified patterns were compared with the qualitative themes to examine how participants' experiences contextualized, elaborated, or differed from the statistical findings. Quantitatively, teacher information literacy, school support, and student cooperation showed significant unique associations with teacher agency, whereas colleague collaboration did not remain significant when the other conditions were considered simultaneously. The qualitative findings provided additional context for understanding how teachers experienced and responded to these personal and contextual conditions through emotional adaptation, professional reflection, and identity negotiation.

Teacher information literacy showed the largest standardized coefficient in the regression model (β = 0.314, *p* < 0.001). The qualitative accounts helped contextualize this pattern. Participants described learning to use technological resources, preparing digital materials, redesigning teaching activities, and adjusting their practices in response to online teaching demands. For example, P1 emphasized the importance of continually learning relevant technologies, while P3 described drawing on online resources and redesigning course content when previous materials were no longer adequate for online instruction. These accounts suggest that information literacy was experienced not simply as technical knowledge but as a practical resource that teachers drew upon when adapting their teaching practices.

School support and student cooperation also showed significant unique associations with teacher agency (β = 0.290 and β = 0.269, respectively, both *p* < 0.001). Qualitative evidence indicated that institutional encouragement, professional development opportunities, teaching resources, and student feedback formed part of the context in which teachers responded to online teaching demands. P1 acknowledged institutional encouragement while also expressing a need for additional professional development and resources. P3 reported that students requested more engaging classroom experiences and additional knowledge beyond the textbook, while P4 emphasized the importance of students' recognition of teaching competence in establishing trust. These accounts add contextual detail to the quantitative associations by illustrating the specific forms of institutional and relational support described by teachers.

A more complex pattern emerged for colleague collaboration. Although colleague collaboration was positively correlated with teacher agency (*r* = 0.417, *p* < 0.001), it did not show a statistically significant unique association in the simultaneous regression model (β = 0.057, *p* = 0.213). The qualitative findings nevertheless indicated that collegial interaction remained meaningful in some teachers' experiences. P2, for example, described collective lesson planning and research-group activities as sources of professional learning and support that helped reduce workload and pressure. This pattern suggests that colleague collaboration may overlap with broader forms of institutional and professional support rather than operating as a distinct correlate once the other conditions are considered simultaneously (Table 9).

**Table 9:** Joint display of quantitative and qualitative findings.

Analytic focus	Statistical result	Qualitative evidence	Integrated interpretation
Teacher information literacy	β = 0.314, *p* < 0.001	Participants described learning technologies, preparing digital materials, using online resources, and redesigning teaching activities.	Teacher information literacy was quantitatively associated with teacher agency, while participants' accounts illustrated how digital information and technological resources were used when adapting teaching practices.
School support	β = 0.290, *p* < 0.001	Participants described institutional encouragement, professional development needs, and access to teaching resources.	Institutional support formed part of the professional context in which teachers enacted agency and adapted to online teaching demands.
Student cooperation	β = 0.269, *p* < 0.001	Participants emphasized student feedback, engagement, and recognition of teaching competence.	Student cooperation provided a relational condition associated with teachers' agency experiences.
Colleague collaboration	β = 0.057, *p* = 0.213; *r* = 0.417, *p* < 0.001	Some participants described collective lesson planning and research-group activities as sources of professional learning and support.	Colleague collaboration was meaningful in teachers' accounts but did not show an independent association with agency after the other conditions were considered simultaneously.
Psychological adaptation	Not quantitatively tested	Emotional adaptation, professional reflection, and identity negotiation were recurring qualitative patterns.	Psychological adaptation provides a qualitative explanatory lens for understanding teachers' reported experiences and responses; it is not established as a mediator or causal mechanism.

Across these findings, psychological adaptation was reflected in how teachers described emotionally responding to uncertainty, reflecting on their practices, and negotiating their professional identities while working within different personal and contextual conditions. These qualitative patterns do not establish psychological adaptation as a mediator between contextual conditions and teacher agency. Rather, they provide an explanatory lens for understanding how teachers experienced and interpreted the conditions associated with agency in the quantitative analysis. Taken together, the two strands therefore support a contextualized interpretation of teacher agency as related to both teachers' personal capacities and their professional environments, while also highlighting variation in how these conditions were experienced by individual teachers.

## 5 Discussion

This study focused on international Chinese language teachers engaged in online teaching. The findings suggest that teacher agency is associated with both teachers' personal capacities and contextual conditions, while teachers' psychological adaptation experiences provide additional insight into how they interpret and respond to changing demands in online teaching. The quantitative findings identified associations between teacher agency and selected personal and contextual conditions, whereas the qualitative findings illustrated teachers' reported experiences of emotional adaptation, professional reflection, and identity negotiation in relation to their agency in online teaching.

### 5.1 Teacher agency and its associated personal and contextual conditions

The study showed that international Chinese language teachers reported an overall mean teacher agency score of 3.45 while teaching online. Among the three dimensions, behavioral agency showed the highest mean score. This finding aligns with ecological perspectives of teacher agency, which define agency as a result of interactions between individuals and their professional environments rather than an inherent personal quality (Biesta and Tedder, 2007; Priestley et al., 2015). Teaching online necessitates the constant interpretation of technology-related challenges, the modification of teaching practices, and decision-making regarding the use of different teaching methods. In this context, behavioral agency was reflected in teachers' efforts to implement and adjust their instructional practices. The qualitative findings provided additional context for understanding these behavioral aspects of teacher agency. Participants described changing teaching methods, exploring digital tools, and adjusting communication strategies in response to the challenges of online teaching. These accounts illustrate how teachers actively adjusted their professional practices rather than simply responding passively to technological change. Identity agency and regulatory agency also constituted important dimensions of teacher agency. Instructional actions of teachers are interlinked to their professional knowledge and self-regulation. Therefore, it can be stated that teacher agency in the context of online teaching is a multi-faceted concept.

### 5.2 Personal and contextual conditions associated with teacher agency

The results indicate that teacher agency is associated with personal resources, institutional contexts, and relational conditions. From an ecological perspective, teacher agency can be understood in relation to both teachers' personal capacities and the opportunities and constraints of their professional environments (Biesta and Tedder, 2007; Priestley et al., 2015).

Among the four conditions examined simultaneously, teacher information literacy showed the largest standardized coefficient in relation to teacher agency (β = 0.314, *p* < 0.001). In this study, teacher information literacy refers to teachers' perceived capacity to access and use digital information and technological resources for teaching.

This interpretation is consistent with previous research suggesting that teachers' digital competence extends beyond technical skills to include the pedagogically meaningful use of digital resources (Falloon, 2020; Redecker, 2017). For international Chinese language teachers, information literacy may therefore represent a relevant personal resource for navigating digitally mediated teaching environments. Recent research on language teacher agency similarly highlights the importance of how teachers perceive and use technological affordances when making pedagogical decisions in digitally mediated environments (Chen, 2022; Lee and Jeon, 2024).

School support also showed a significant unique association with teacher agency. This finding is consistent with ecological perspectives emphasizing that agency is enacted within institutional contexts. Supportive contexts give teachers access to technology, professional learning, and institutional recognition. Such institutional resources may provide conditions that support teachers' professional learning and pedagogical adjustment (Opfer and Pedder, 2011). The qualitative findings add nuance to this association: participants distinguished between general institutional encouragement and more specific forms of support, such as professional development opportunities, guidance, and teaching resources. Similarly, studies of technology-mediated language teaching have shown that institutional training and material support form part of the structural conditions within which teachers enact agency (Lee and Jeon, 2024). These findings reinforce the view that school support is not merely a background condition but part of the professional environment within which teachers make pedagogical choices.

Student cooperation also showed a significant unique association with teacher agency. In online teaching contexts, student participation and feedback form an important part of teachers' relational environment. The qualitative findings further showed that participants attended to student feedback and responses when evaluating and adjusting their teaching practices. Recent research also suggests that teacher–student interaction in online settings is reciprocal: teachers exercise agency in attempting to sustain engagement, while students' responses simultaneously become part of the conditions to which teachers adapt their practices.

Although colleague collaboration was positively correlated with teacher agency (*r* = 0.417, *p* < 0.001), it did not show a statistically significant unique association in the simultaneous regression model (β = 0.057, *p* = 0.213). This pattern suggests that the association between colleague collaboration and teacher agency may overlap with other aspects of the professional environment when the four conditions are considered simultaneously. Nevertheless, the absence of a significant unique coefficient does not imply that collegial interaction was unimportant in teachers' professional experiences. For example, P2 described collective lesson planning and research-group activities as sources of professional learning and support that helped reduce workload and professional pressure. This finding differs somewhat from studies in which collegial collaboration has been closely linked to the enactment of teacher agency. For example, Brodie (2021) showed that collaboration within professional learning communities can provide conditions for teachers to engage agentically in professional learning, while Shavard (2022) demonstrated that collaborative lesson planning can create spaces for professional agency. However, other research suggests that the relationship between collaboration and agency is conditional rather than uniform; Lau (2021), for example, found that teachers' agency in collaborative settings varied across ecological and organizational boundaries. The present findings therefore suggest that colleague collaboration may remain professionally meaningful even when its unique statistical association with teacher agency is attenuated after other contextual conditions are considered simultaneously.

Overall, these findings indicate that teacher agency is associated with a combination of personal and contextual conditions. At the same time, the quantitative associations alone do not capture how teachers experienced and interpreted these conditions in their professional practice. The qualitative findings provide a complementary explanatory lens by illustrating how teachers described emotional adaptation, professional reflection, and identity negotiation in relation to their experiences of agency in online teaching.

### 5.3 Psychological adaptation as an explanatory lens on teachers' agency experiences

The qualitative findings identified three recurring patterns in teachers' psychological adaptation experiences: emotional adaptation, professional reflection, and identity negotiation. These patterns provide a qualitative lens for understanding how teachers described experiencing, interpreting, and responding to changing professional conditions in relation to their agency. Psychological adaptation was not examined quantitatively as a mediating variable in this study; therefore, the qualitative findings should not be interpreted as evidence of a mediating or causal mechanism linking personal or contextual conditions to teacher agency.

The first recurring pattern concerned emotional adaptation. Online education comes with elements of uncertainty and technology issues, and it can challenge the norms of teacher–student interaction, creating emotional tension for teachers. However, participants described efforts to regulate negative emotions, manage uncertainty, and reconsider their experiences of online teaching. Their accounts suggest that emotional adaptation involved attempts to remain professionally engaged while responding to uncertainty and pressure in online teaching. This interpretation is consistent with recent research showing that teachers' emotional experiences and agency are closely intertwined in online teaching. Tao et al. (2024), for example, found that teachers experienced complex trajectories of positive and negative emotions during online teaching and that these emotional experiences were closely connected to their enactment of agency. Similarly, Janes and Chen (2024) showed that emotion regulation, professional identity, and agency were dynamically interconnected in the experiences of a novice Chinese language teacher during emergency remote teaching. These findings support the interpretation of emotional adaptation in the present study as part of teachers' reported experiences of responding to changing teaching demands rather than as an independent psychological mechanism. The second recurring pattern was professional reflection. Participants described critically reviewing their online teaching practices and evaluating different instructional approaches. Participants' accounts indicated that reflection involved reconsidering available technological and institutional resources in relation to their instructional needs and goals. The third recurring pattern concerned identity negotiation. Participants described reconsidering their professional roles and responsibilities in the context of technology-mediated teaching. Their accounts illustrate how questions of professional identity were intertwined with teachers' understandings of what it meant to be an effective educator in online settings. The identity-related findings also correspond with research showing that changes in teaching context can prompt teachers to reconsider and reconstruct their professional identities. Zhang and Hwang (2023), for example, documented context-sensitive processes of language teacher identity reconstruction during the transition to online teaching. However, such identity reconstruction should not be understood as following a uniform developmental trajectory, as teachers' responses may vary according to prior experience, emotional conditions, institutional contexts, and available technological affordances. This variation is consistent with the differences observed across the qualitative cases in the present study and supports interpreting psychological adaptation as context-dependent and heterogeneous rather than as a fixed pathway through which teacher agency develops.

Taken together, these findings complement ecological perspectives of teacher agency by drawing attention to how teachers experience and interpret professional conditions in online teaching. The qualitative accounts showed that teachers described interpreting and responding to these conditions through emotional adaptation, professional reflection, and identity negotiation. In this view, psychological adaptation provides a qualitative explanatory lens for understanding how teachers experience, interpret, and respond to changing professional conditions.

In addition, it is important to distinguish teacher agency conceptually from related psychological constructs such as self-efficacy.

Self-efficacy generally concerns teachers' beliefs about their capability to perform particular tasks successfully, whereas teacher agency concerns teachers' capacity to make purposeful choices and enact professional actions within particular social and professional conditions.

In the present study, psychological adaptation is therefore understood more broadly than an emotional response to the difficulties of online teaching; the qualitative findings reflected teachers' experiences of emotional adaptation, professional reflection, and identity negotiation.

The findings therefore suggest that teachers' agency experiences can be understood in relation to both their personal and contextual conditions and their reported psychological adaptation experiences. Teachers facing broadly similar professional demands may nevertheless describe different ways of interpreting and responding to those demands. This perspective provides a more contextualized understanding of teacher agency in digitally mediated teaching by considering psychological adaptation as a complementary qualitative lens rather than as a tested causal mechanism.

### 5.4 Theoretical and practical implications

The findings of this study have multiple practical implications for promoting agentive teaching practices in online education. First, educational institutions should not only offer technological resources but also work on the development of teachers' information literacy skills. While providing access to teaching technologies creates unique opportunities for online teaching, teachers need to possess relevant knowledge and skills for efficient application of these tools in their teaching. Thus, teacher training programs should focus on both technical knowledge and reflective practices that may help teachers use technology effectively.

Second, institutional support may usefully consider teachers' reported psychological adaptation experiences alongside their professional development needs. The evidence shows that support from educational institutions is significantly associated with teacher agency, indicating the relevance of an enabling organizational environment for teachers' professional practice. Educational institutions can provide continuous professional development, opportunities for cooperation with peers, and relevant technical assistance to support teachers in adapting to the changing teaching environment.

Third, the relational aspect of online education should be taken into consideration. Student cooperation showed a significant positive association with teacher agency. This is why proper online communication and establishment of supportive relationships between teachers and students may be relevant to teachers' professional involvement. At the same time, the possibilities of collaboration and experience sharing among teachers should be extended, as professional communities may provide opportunities for reflection and professional learning in digital environments. Finally, the analysis reiterates the necessity of addressing the psychological component of teacher development. Professional development programs should provide opportunities for teachers to reflect on their experiences, navigate changes in professional identity, and develop strategies for coping with emotional challenges. Support for teacher agency should therefore consider not only teachers' professional conditions but also their capacity to reflect on and respond to changes in education.

## 6 Conclusion

In this research, the associations between selected personal and contextual conditions and teacher agency among international Chinese language teachers engaged in online teaching were investigated, together with teachers' psychological adaptation experiences in relation to teacher agency. This study used an explanatory sequential mixed-method approach, integrating quantitative data from 312 teachers with qualitative data from four cases.

According to the findings, the overall mean score for teacher agency was 3.45, with behavioral agency showing the highest mean score among the three dimensions.

The quantitative results showed that teacher information literacy, school support, and student cooperation were significantly associated with teacher agency, while the qualitative findings identified emotional adaptation, professional reflection, and identity negotiation as recurring patterns in teachers' psychological adaptation experiences in the changing online teaching environment.

The present study contributes to research on teacher agency by examining agency in relation to personal and contextual conditions in online teaching. The findings suggest that understanding teacher agency requires attention to both the personal and contextual conditions associated with agency and teachers' reported experiences of interpreting and responding to these conditions. By considering psychological adaptation as a qualitative explanatory lens, the present research provides additional insight into how teachers experience and respond to changing professional conditions.

The research also has practical implications for teacher training in the field of digital education. To support teacher agency, merely having technological infrastructure and resources is not enough. Educational institutions should provide support for teachers' information literacy, professional reflection, identity-related experiences, and adaptation to changing teaching conditions. Such support may help teachers respond to continuing changes in technology-mediated teaching.

There exist various limitations that must be recognized. Firstly, although the sample was adequate for the CFA and regression analyses, the participants were selected from particular online educational settings, limiting the transferability of the results obtained. Future studies may concentrate on larger and more heterogeneous sample sizes which may help in producing results which could be applicable to different cultures and educational institutions. Secondly, the qualitative part of the study included four purposively selected teachers, allowing in-depth exploration of individual experiences but not broader representation. Future research could use longitudinal designs to examine how psychological adaptation experiences and teacher agency change over time. Finally, while the qualitative findings highlight the relevance of psychological adaptation experiences, future quantitative studies could examine associations among personal and contextual conditions, psychological adaptation, and teacher agency.

In addition, the cross-sectional and self-report nature of the quantitative data does not permit causal conclusions about the relationships between teacher agency and the conditions examined. The findings may also be subject to common method bias and socially desirable responding.

## Data Availability

The datasets presented in this article are not readily available due to privacy and ethical considerations. The raw data are not publicly available. However, de-identified quantitative data may be provided by the corresponding author upon reasonable request, subject to applicable ethical and confidentiality considerations. Requests to access the datasets should be directed to Yi Wu, wuyi19871987@gmail.com.
